# Emerging biotechnologies and biomedical engineering technologies for hearing reconstruction

**DOI:** 10.1002/SMMD.20230021

**Published:** 2023-10-27

**Authors:** Yangnan Hu, Le Fang, Hui Zhang, Shasha Zheng, Menghui Liao, Qingyue Cui, Hao Wei, Danqi Wu, Hong Cheng, Yanru Qi, Huan Wang, Tao Xin, Tian Wang, Renjie Chai

**Affiliations:** ^1^ State Key Laboratory of Digital Medical Engineering Department of Otolaryngology Head and Neck Surgery Zhongda Hospital School of Life Sciences and Technology Advanced Institute for Life and Health Jiangsu Province High‐Tech Key Laboratory for Bio‐Medical Research Southeast University Nanjing China; ^2^ Co‐Innovation Center of Neuroregeneration Nantong University Nantong China; ^3^ Department of Neurology The China‐Japan Union Hospital of Jilin University Changchun Jilin China; ^4^ Department of Otolaryngology Head and Neck Surgery Affiliated Drum Tower Hospital of Nanjing University Medical School Jiangsu Provincial Key Medical Discipline Nanjing China; ^5^ The Eighth Affiliated Hospital Sun Yat‐Sen University Shenzhen China; ^6^ Department of Neurosurgery The First Affiliated Hospital of Shandong First Medical University & Shandong Provincial Qianfoshan Hospital Jinan China; ^7^ Medical Science and Technology Innovation Center Shandong First Medical University and Shandong Academy of Medical Sciences Jinan China; ^8^ Department of Otolaryngology‐Head and Neck Surgery Stanford University School of Medicine Stanford California USA; ^9^ Department of Otolaryngology‐Head and Neck Surgery The Second Xiangya Hospital Central South University Changsha Hunan Province China; ^10^ Department of Otolaryngology Head and Neck Surgery Sichuan Provincial People's Hospital University of Electronic Science and Technology of China Chengdu China; ^11^ Institute for Stem Cell and Regeneration Chinese Academy of Sciences Beijing China; ^12^ Beijing Key Laboratory of Neural Regeneration and Repair Capital Medical University Beijing China

**Keywords:** drug delivery, electrical stimulation, gene therapy, hearing reconstruction, organoid

## Abstract

Hearing impairment is a global health problem that affects social communications and the economy. The damage and loss of cochlear hair cells and spiral ganglion neurons (SGNs) as well as the degeneration of neurites of SGNs are the core causes of sensorineural hearing loss. Biotechnologies and biomedical engineering technologies provide new hope for the treatment of auditory diseases, which utilizes biological strategies or tissue engineering methods to achieve drug delivery and the regeneration of cells, tissues, and even organs. Here, the advancements in the applications of biotechnologies (including gene therapy and cochlear organoids) and biomedical engineering technologies (including drug delivery, electrode coating, electrical stimulation and bionic scaffolds) in the field of hearing reconstruction are presented. Moreover, we summarize the challenges and provide a perspective on this field.

## Graphical abstract

Biotechnologies and biomedical engineering technologies provide new hope for the treatment of auditory diseases, which utilizes biological strategies or tissue engineering methods to achieve drug delivery and the regeneration of cells, tissues, and even organs. Here, the advancements in the applications of biotechnologies and biomedical engineering technologies in the field of hearing reconstruction are presented. Finally, the authors summarize the challenges and provide a perspective on this field.

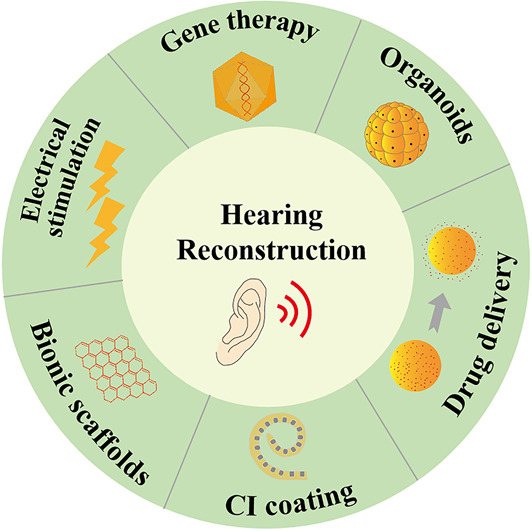

**Key points**

- Recent advancements in biotechnologies and biomedical engineering technologies for hearing reconstruction are presented.
- Biomedical engineering technologies including drug delivery, electrode coating, electrical stimulation and bionic scaffolds for hearing reconstruction are emphasized.
- The future challenges and development directions of biotechnologies and biomedical engineering technologies in the field of hearing are presented.

## 1 INTRODUCTION

Hearing impairment is a common sensory defect disease.^1^, ^2^ In 2021, the number of people with hearing disabilities worldwide will be 278 million, which is 4.6% of the total global population. With the abuse of ototoxic drugs, noise and environmental pollution, and aging populations, the prevalence of hearing loss is rising yearly. Therefore, deafness has become a global health problem that affects social politics and the economy. The damage and loss of auditory cells such as hair cells (HCs) and spiral ganglion neurons (SGNs) and the degeneration of neurites of SGNs are the core causes of sensorineural hearing loss.^3^, ^4^, ^5^, ^6^, ^7^ Despite the rapid development of medical technology, there is no safe, feasible and effective therapeutic method to repair the damage of auditory. Therefore, many researchers have focused on the reconstruction of hearing using new biological strategies and engineering approaches, which have provided new hope for the treatment of auditory diseases. In existing studies, researchers have delivered genes or therapeutic drugs to the inner ear and induced the regeneration of cells in the cochlea and the formation of cochlea organoids, which are conducive to treating hearing loss.

**FIGURE 1:**
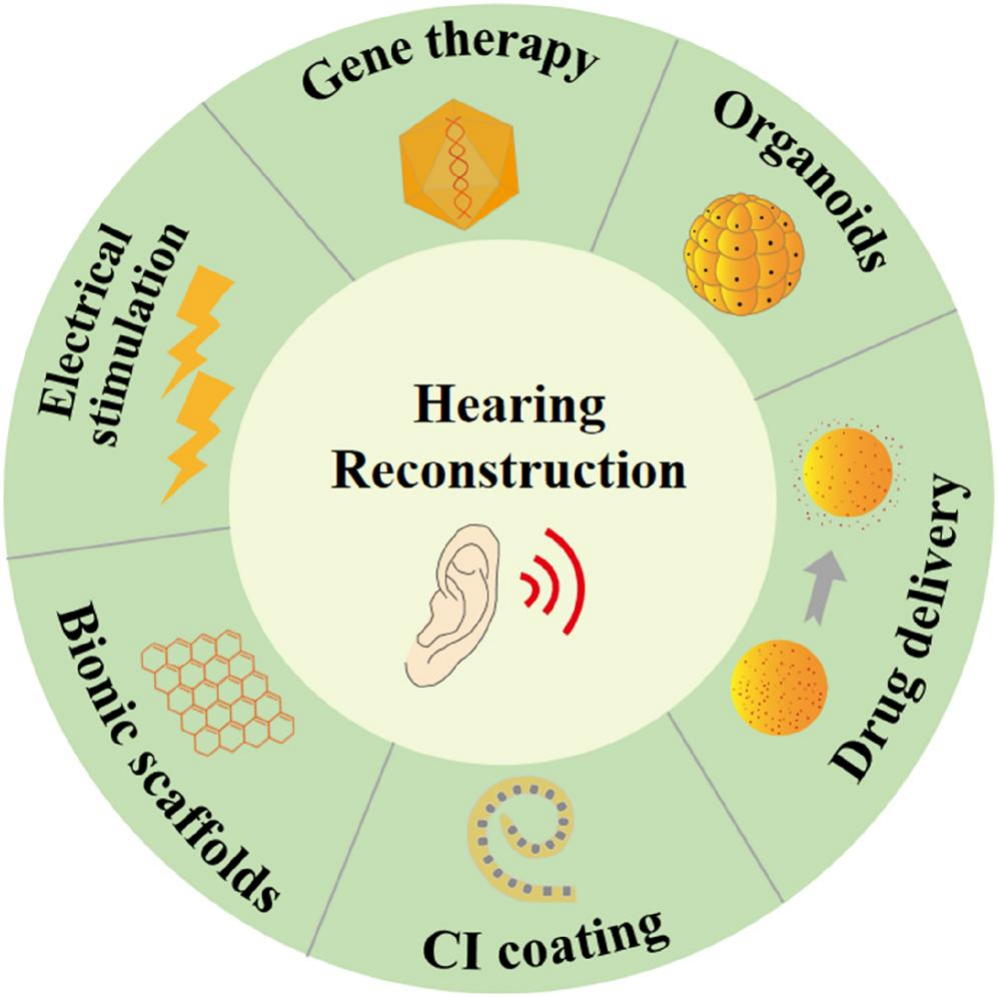
Schematic illustration of different biotechnologies and biomedical engineering technologies for hearing construction.

In this paper, we present a concise summary of the latest advances in biotechnologies and biomedical engineering technologies for repairing damaged auditory structures (Figure 1). Firstly, we will give a brief description of the structure of the ear, as the physiological structure determines the development of biomedical engineering technologies. After that, we will focus on emerging biotechnologies and biomedical engineering technologies for hearing reconstruction, including gene therapy, cochlea organoids, inner ear drug delivery, cochlear implant (CI) electrode coating, electrical stimulation, and bionic scaffolds. Finally, we will discuss the recent challenges and give a future outlook on these technologies in the auditory system.

## 2 ANATOMY OF THE MAMMALIAN EAR

**FIGURE 2:**
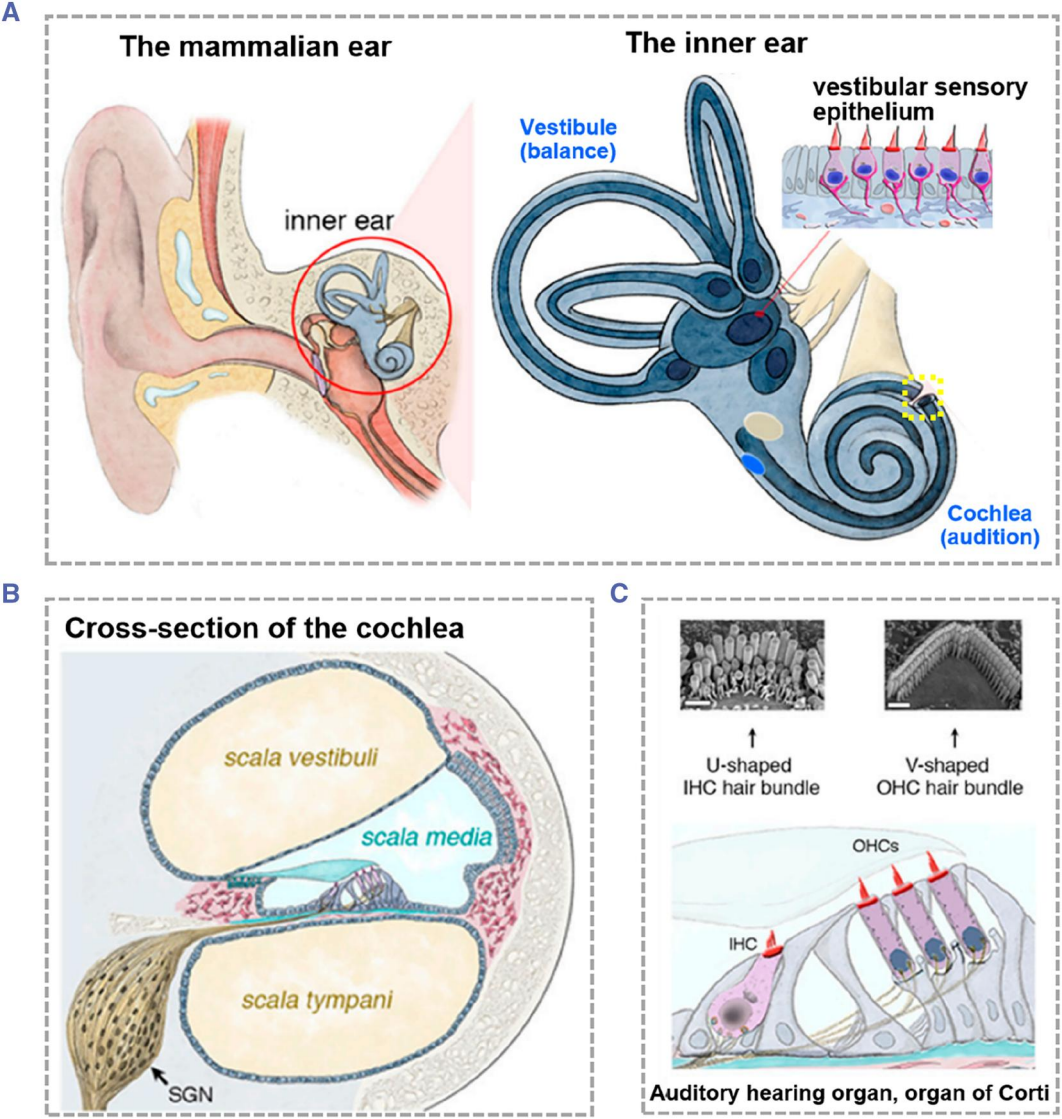
Anatomy of the inner ear in mammals. (A) The location and structure of the inner ear. (B) The cross‐section of the cochlea displaying three fluid‐filled longitudinal tubes. (C) The organ of Corti. Reproduced under terms of the CC‐BY license.^13^ Copyright 2020, The Authors, published by MDPI.

The external ear, middle ear, and inner ear are the three anatomical divisions of the ear. The external ear contains three parts: the auricle, the external ear canal and the tympanic membrane. The external ear canal is a passage from the auricle to the tympanic membrane, which is an oval translucent membrane located between the external ear canal and the tympanic cavity.^8^, ^9^ The middle ear consists of the tympanic cavity, pharyngotympanic tube, tympanic antrum, and mastoid process.^5^, ^10^ The inner ear is surrounded by the temporal bone, and is also called the labyrinth due to its complex and precise structure. The inner ear is connected to the brain stem and the auditory nerve through the inner auditory canal, transmitting sound signals to the auditory center of the brain together.^11^, ^12^ As shown in Figure 2A,B, the inner ear includes the cochlea, the vestibular organ, and three semicircular canals, with the vestibular organ and semicircular canals responsible for postural balance and the cochlea responsible for hearing.^14^

The cochlea is an auditory organ with the characteristic curling shape of a snail, and the number of turns of its spirals varies among species.^15^ The cochlea consists of three longitudinal tubes filled with fluid, it is the scala vestibule, scala media, and scala tympani arranged from top to bottom (Figure 2B). The scala tympani ends in a semi‐permeable round window membrane (RWM). The organ of Corti consists of HCs and supporting cells (SCs) located on the basilar membrane on the side of the scala media, and the HCs include a row of highly organized inner HCs (IHCs) and three rows of outer HCs (OHCs) (Figure 2C). The sensory IHCs respond to sound stimuli and convert them into electrical signals on nerve fibers, which are also amplified by the OHCs. The resulting nerve impulses transmit along the auditory conduction system of the brainstem to the auditory cortex to produce hearing.

## 3 BIOTECHNOLOGIES FOR HEARING RECONSTRUCTION

### 3.1 Gene therapy

About 1 in 500 newborns suffer from congenital hearing impairment, and more than 50% are hereditary.^16^, ^17^, ^18^ Although more than 150 genes related to hearing loss have been identified, there are no effective strategies for treatment in the clinic. Mutations that affect the function of HCs, SCs, or stria vascularis are the three main types of mutations that cause severe hearing loss. Hereditary deafness mainly includes syndromic and nonsyndromic types. Syndrome mutations lead to hearing loss and other dysfunctions, such as cardiac arrhythmia. Nonsyndromic deafness affects only the ear and includes 43 genes carrying autosomal recessive mutations and 25 genes carrying dominant mutations.^1^

**FIGURE 3:**
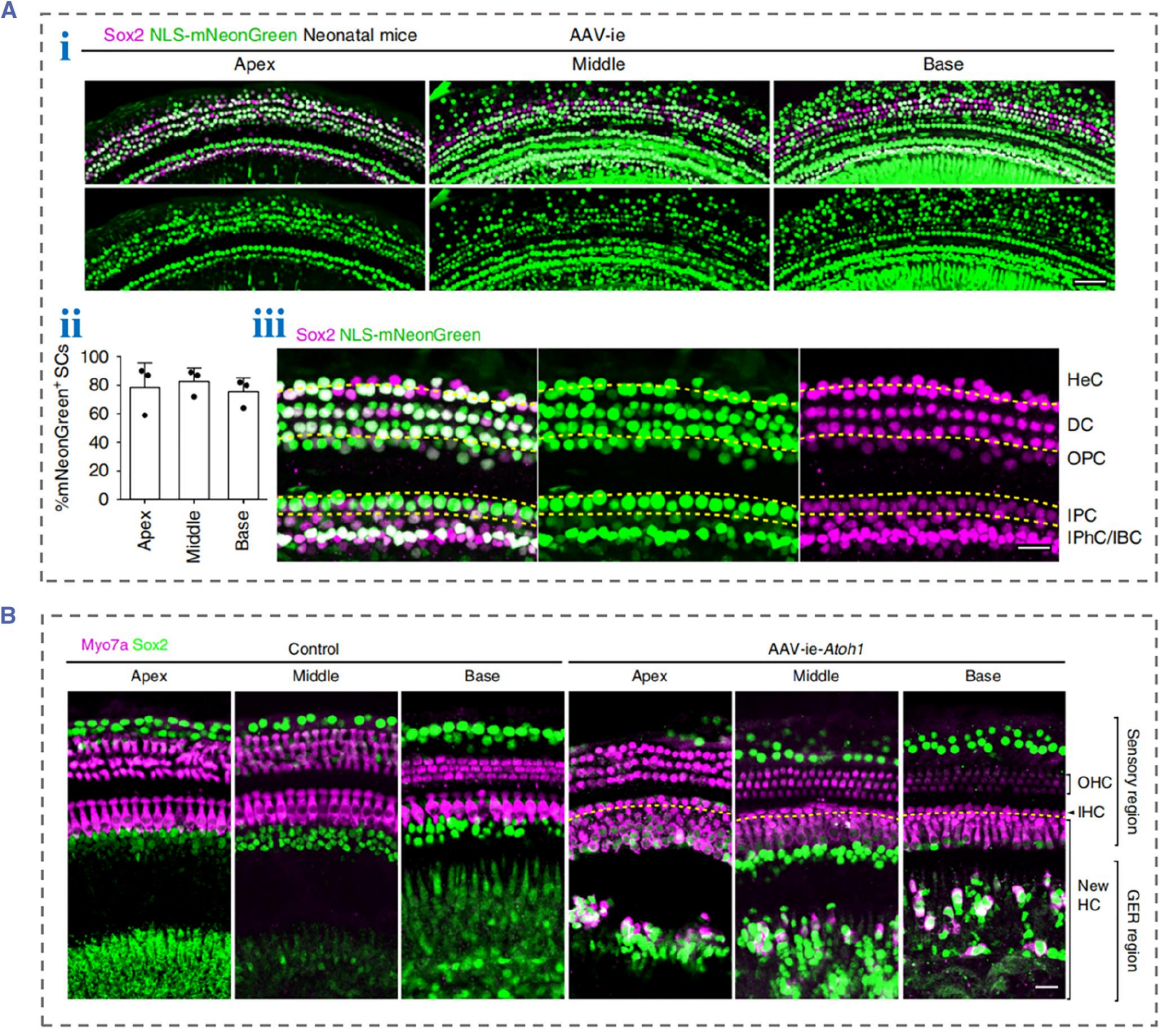
(A) (i) Confocal images showing AAV‐ie targeted SCs in the cochlea. Scale bar: 50 μm. (ii) Statistics of SCs expressing NLS‐mNeonGreen per 100 μm in (i). (iii) Confocal images of different types of SCs in apical turn of cochleae, including HeC, DC, OPC, IPC, and IBC. Scale bar: 20 μm. (B) Confocal projection images showing AAV‐ie‐Atoh1‐induced regeneration of HCs in vivo with stereocilia. Scale bar: 20 μm. Reproduced under terms of the CC‐BY license.^22^ Copyright 2019, The Authors, published by Springer Nature. AAV, adeno‐associated virus; DC, Deiters cells; HeC, Hensen's cells; IBC, inner border cells; IPC, inner pillar cells; NLS‐mNeonGreen, nuclear mNeonGreen; OPC, outer pillar cells; SCs, supporting cells.

With the innovation and development of biomedical technologies, gene therapy has been shown to be effective in animal models of deafness and is gradually entering the clinic. Adeno‐associated virus (AAV) is one of the most commonly used vectors in the field of gene therapy and has great application prospects in gene therapy for hearing diseases. In 2017, Landegger et al. reported an artificially designed AAV vector Anc80L65.^19^ Compared with the conventional serotype AAV, Anc80L65 significantly improved the infection efficiency of the IHCs and OHCs in juvenile mice. Subsequently, a study reported that Anc80L65 had an almost 100% infection rate in the IHCs of adult mice but a meager infection rate in the OHCs.^20^ In 2019, another novel AAV, AAV2.7m8, was discovered and reported.^21^ Compared with Anc80L65, AAV2.7m8 with a high titer showed stronger infective efficiency on HCs. Moreover, AAV2.7m8 also infected inner phalangeal cells and inner border cells. However, both AAV2.7m8 and Anc80L65 have low infection rates in other SCs, which is their disadvantage in gene therapy targeting SCs. Surprisingly, by modifying the AAV capsid protein, Tan et al. developed AAV‐ie, an AAV vector with high infective efficiency to all cells in the cochlea.^22^ An AAV reporter genome expressing nuclear mNeonGreen (NLS‐mNeonGreen) was packaged using the AAV‐ie vector. Their results demonstrated that the infection rates of AAV‐ie to cochlear SCs, including Hensen's cells, Deiters cells, pillar cells, IPhCs, and IBCs, were significantly higher than those of AAV2.7m8 and Anc80L65, reaching about 80%, which indicated its potential in the treatment of hearing diseases (Figure 3A). Moreover, they used AAV‐ie to introduce the Atoh 1 gene into the cochlea of juvenile mice, achieving the regeneration of IHCs by AAV vector for the first time (Figure 3B).

### 3.2 Cochlear organoids

Organoids provide promising platforms for both development and regenerative research.^23^, ^24^ Tissue‐specific stem cells have been found in the sensory epithelium of the cochlea of postnatal mice, which are LGR5^+^ SCs and have the ability to proliferate and differentiate into HCs and SCs.^25^, ^26^, ^27^ In recent years, cochlear organoids have been induced from numerous stem cells including embryonic stem cells (ESCs) and cochlear progenitors, etc.^28^, ^29^, ^30^, ^31^, ^32^ The cell composition and physiological characteristics of cultured cochlear organoids are highly similar to those of organs in vivo, so they can be used as ideal models for the study of organ development, function and disease.^29^

**FIGURE 4:**
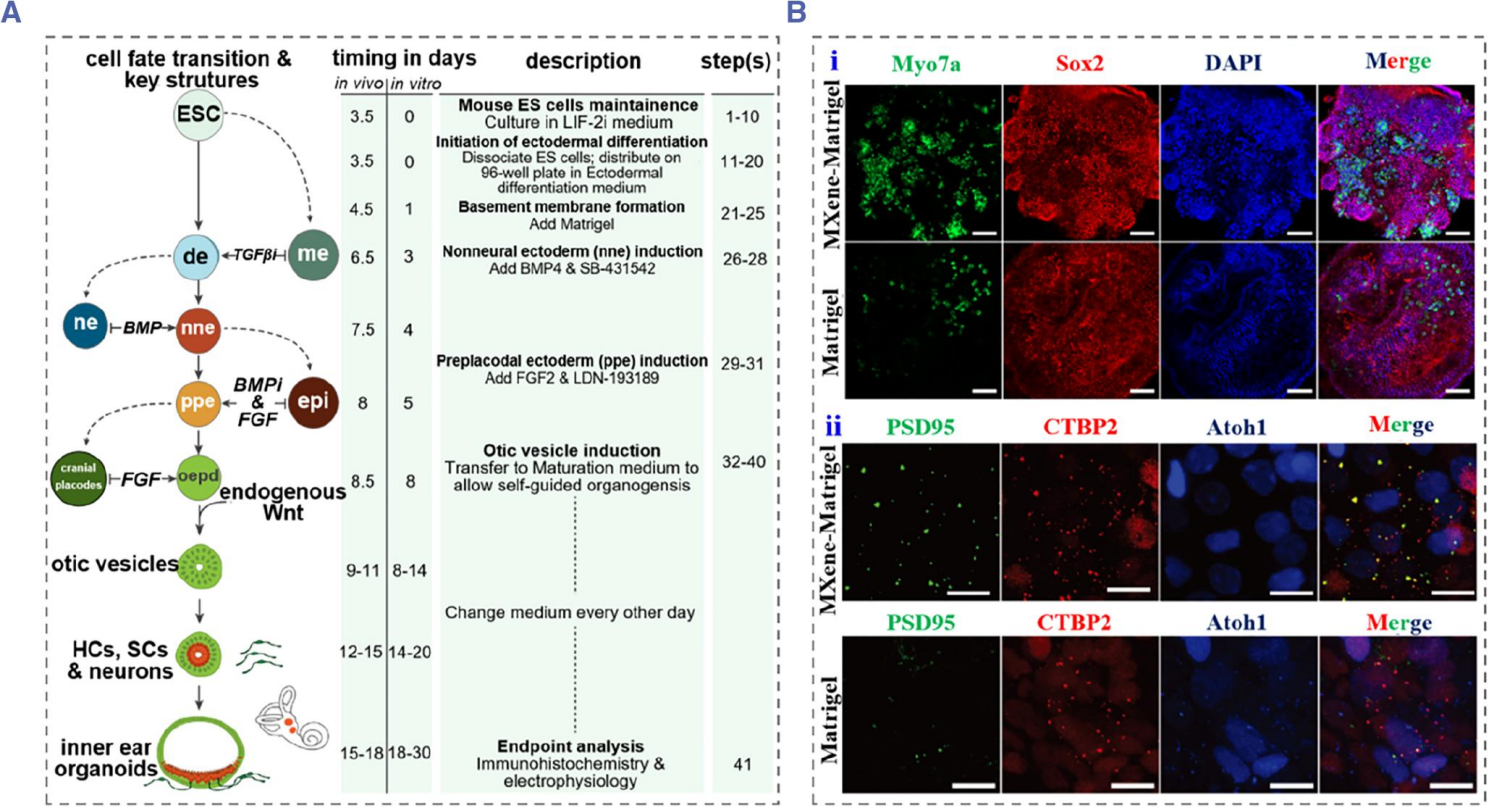
(A) Overview of the inner ear induction method. Reproduced with permission.^30^ Copyright 2014, Springer Nature. (B) (i) Immunofluorescence images of cochlear organoids. Myo7a (green) is a marker of HCs, Sox2 (red) is a marker of SCs, and DAPI (blue) represents the nuclei of cells. Scale bar: 50 μm. (ii) Confocal images of co‐localization of CTBP2^+^ and PSD95^+^ puncta in cochlear organoids. PSD95 (green) is a postsynaptic marker, CTBP2 (red) is a pre‐synaptic marker, and Atoh1 (blue) represents HCs. Scale bar: 10 μm. Reproduced under terms of the CC‐BY license.^33^ Copyright 2022, The Authors, published by John Wiley and Sons. DAPI, 2‐(4‐Amidinophenyl)‐6‐indolecarbamidine dihydrochloride; HeC, Hensen's cells; SCs, supporting cells.

In a 3D matrigel culture system, Koehler and Hashino reported a method to induce the differentiation of ESCs into inner ear organoids containing HCs in a 3D matrigel culture system, as shown in Figure 4A.^30^ Specifically, mouse ESCs were aggregated in a culture medium containing factors that induce epithelialization. In the first 14 days, the preplacodal ectoderm, embryonic nonneural ectoderm, and otic vesicle epithelia were induced by a series of proteins and small molecules with precise timing control. Finally, they developed the ESC‐derived epithelia containing HCs, SCs, and sensory‐like neurons. Importantly, these neurons could form synapse‐like structures with the differentiated HCs. Moreover, another study by Koehler and his colleagues in 2017 reported a method for differentiating hPSCs to inner ear organoids with functional HCs. They obtained otic vesicle‐like structures from a single stem‐cell aggregate by modulating transforming growth factor, bone morphogenetic protein, fibroblast growth factor, and Wnt signaling under 3D culture conditions.^29^ Their protocols provide a potential method to form the inner ear sensory tissue in vitro. Recently, Zhang et al. reported a 3D culture system by incorporating conductive Ti_3_C_2_TxMXene into matrigel (MXene‐Matrigel) and studied its influence on the differentiation and development of cochlear organoids (Figure 4B).^33^ The fabricated MXene‐Matrigel 3D system exhibited great cytocompatibility and promoted the differentiation and maturation of HCs from cochlear organoids. They also found that the regenerated HCs were comparable to native HCs by electrophysiological analysis. Furthermore, the results showed that the prepared MXene‐Matrigel facilitated HC differentiation by activating mTOR signaling. Finally, they described a co‐culture system of inner ear organoids and modiolus, revealing the innervations of the modiolus and regenerated HCs. Moreover, the formation efficiency of synaptic connections between organoid HCs and SGNs in the MXene‐Matrigel system was significantly higher than that of pure matrigel. Therefore, cochlear organoids provide a promising platform for the development of drugs or genetic carrier tools to benefit hearing impaired patients in the future.

## 4 BIOMEDICAL ENGINEERING TECHNOLOGIES FOR HEARING RECONSTRUCTION

### 4.1 Inner ear drug delivery

Systemic and local administration are the main approaches to deliver drugs into the inner ear.^34^, ^35^ Compared to local drug delivery, systemic administration has some limitations due to crossing the blood‐labyrinth barrier.^36^, ^37^ Therefore, researchers are increasingly focusing on the local administration in the inner ear. Over the past few decades, various drug delivery systems and techniques have been developed and studied. Among them, nanoparticle‐based delivery systems,^38^, ^39^, ^40^, ^41^ microsphere‐based delivery systems,^42^ and pump/catheter‐based delivery systems^43^, ^44^, ^45^ have been the most extensively presented.

**FIGURE 5:**
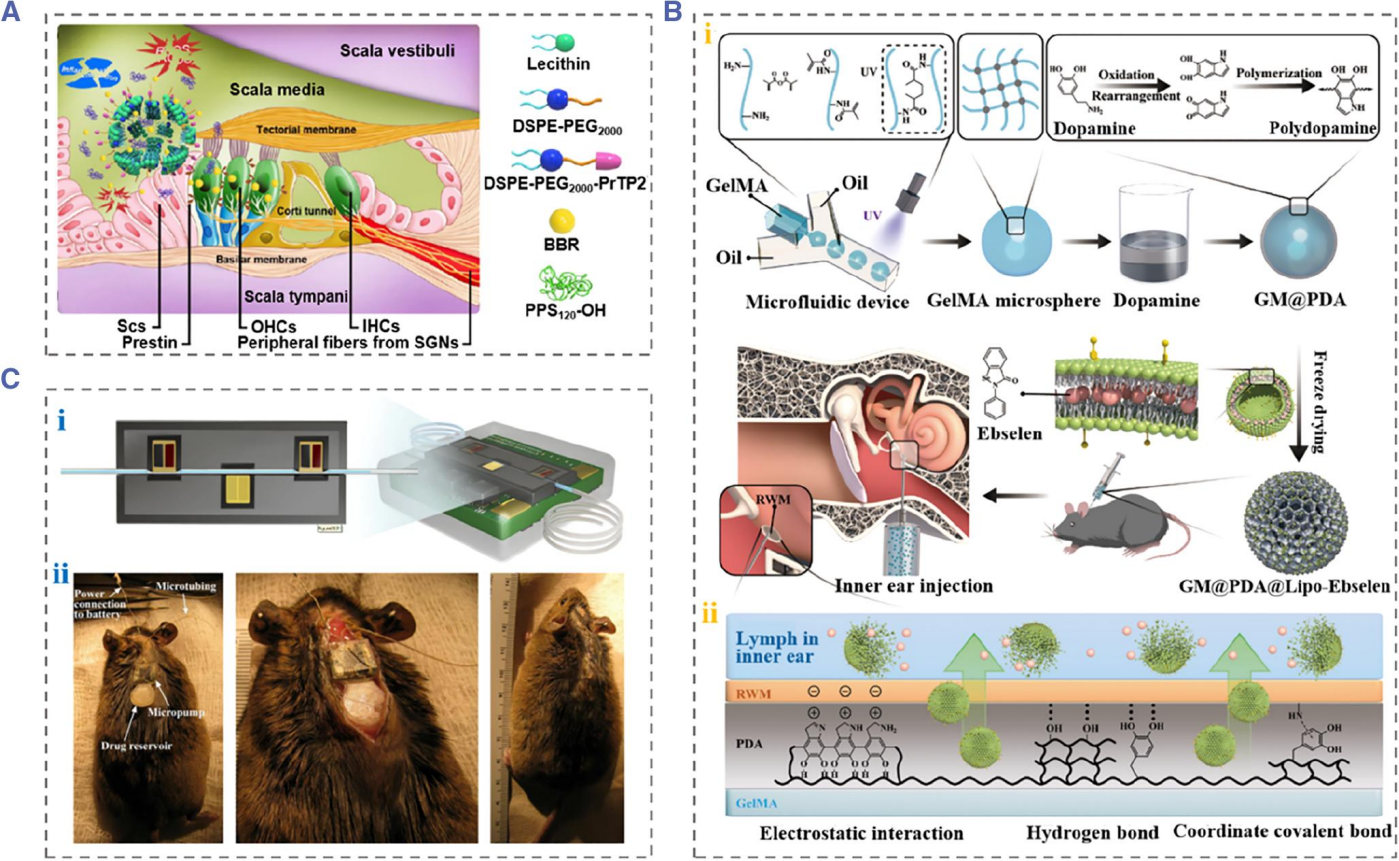
Drug delivery system for hearing loss treatment. (A) Schematic of ROS‐responsive nanoparticles delivering BBR to the inner ear. Reproduced with permission.^50^ Copyright 2021, American Chemical Society. (B) (i) The preparation process of GM@PDA@Lipo‐Ebselen and the diagram of injecting microspheres onto RWM. (ii) Diagram of the process of drugs entering into the inner ear through RWM. Reproduced with permission.^42^ Copyright 2022, John Wiley and Sons. (C) (i) Peristaltic micropump for inner ear administration. (ii) Photographs of a mouse after subcutaneous implanting a peristaltic micropump 1 month. Reproduced with permission.^51^ Copyright 2019, Elsevier. BBR, berberine; ROS, reactive oxygen species; RWM, round window membrane.

Delivering therapeutic drugs by nanoparticles has become a promising method to treat inner ear disorders by virtue of their small size, high loading efficiency and surface functionalization.^46^ Many types of nanoparticles have been developed and used for inner ear drug delivery, including inorganic nanoparticles, lipids, and polymers, etc.^46^, ^47^, ^48^, ^49^ For example, Zhao et al. constructed the reactive oxygen species (ROS)‐responsive nanoparticles loading berberine (BBR), named PL‐PPS/BBR and investigated their application in noise‐induced hearing loss (NIHL) therapy.^50^ The PL‐PPS/BBR were obtained by the self‐assembly of BBR, poly (propylene sulfide)_120_‐OH (PPS_120_‐OH), lecithin, DSPE‐PEG_2000_, and PrTP2‐conjugated DSPE‐PEG_2000_, as shown in Figure 5A. The PPS_120_‐OH in the nanoparticles underwent a structural transformation when exposed to a ROS environment in the inner ear, thereby releasing BBR with antioxidant and anti‐inflammatory effects. The in vivo experimental results demonstrated that the OHCs were protected and the hearing level of the NIHL guinea pig was improved significantly.

Hydrogel microspheres are excellent drug delivery carriers with particle size at the micron level, which can be obtained by electro‐hydrodynamic spraying, batch emulsion, photolithography, and microfluidic techniques.^52^, ^53^, ^54^ Compared with other technologies, microfluidic technology allows mass production of monodisperse microspheres with specific diameters in batches and microspheres with various complex functions. For example, Chen et al. fabricated methacrylate gelatin microspheres using microfluidics, coated them with a polydopamine (PDA) layer, and loaded with Ebselen liposomes (GM@PDA@Lipo‐Ebselen) (Figure 5B).^42^ Benefiting from the great adhesive properties of PDA, the prepared microspheres could be easily injected onto the RWM and closely adhered to the surface of the RWM. Therefore, the loaded drugs could be kept in the middle ear longer, which allowed more drugs to enter the inner ear and finally treat NIHL effectively.

Additionally, numerous pump‐/catheter‐based systems have also been developed and widely applied for sustained drug delivery in many fields.^55^, ^56^, ^57^ These pumps allow precise control of low infusion rates and continuous drug delivery for a long time. For example, researchers have constructed an implantable peristaltic micropump to achieve accurate drug delivery to the RWM using 3D‐printing technology.^51^ Figure 5C displays a schematic diagram of the peristaltic micropump and the actuation mechanism. The micropump was deep‐rooted into the animal scalp and the microtubule was inserted into their middle ear. Their in vivo results demonstrated that the micropump system could infuse sodium salicylate into the RWM at a rate of 50 nL/min for 20 min. Moreover, there was no inflammation or infection a month after the micropump was implanted.

### 4.2 CI electrode coating

Conventional hearing aids can be used to restore hearing in patients with mild and moderate hearing loss, while CI can be used for recovering hearing in patients with severe to profound sensorineural hearing loss.^58^, ^59^ Although CIs offer great hope to many patients, it has been found that patients implanted with CIs are likely to lose residual hearing within months.^60^ The main reasons are the loss of auditory HCs caused by the surgical insertion of electrodes into the scala tympani, and the high impedance of the electrodes after mechanical trauma.^61^, ^62^, ^63^, ^64^ The latter is induced by fibrosis in the cochlea and new ear bones so that the host's response time to the electrical signal is prolonged.^61^, ^65^ Therefore, researchers have focused on the CI electrode surface coating prepared by different biomaterials to deliver neurotrophins,^66^, ^67^, ^68^ genes,^69^ or cells^70^, ^71^, ^72^ to improve the microenvironment after implantation and hearing.

**FIGURE 6:**
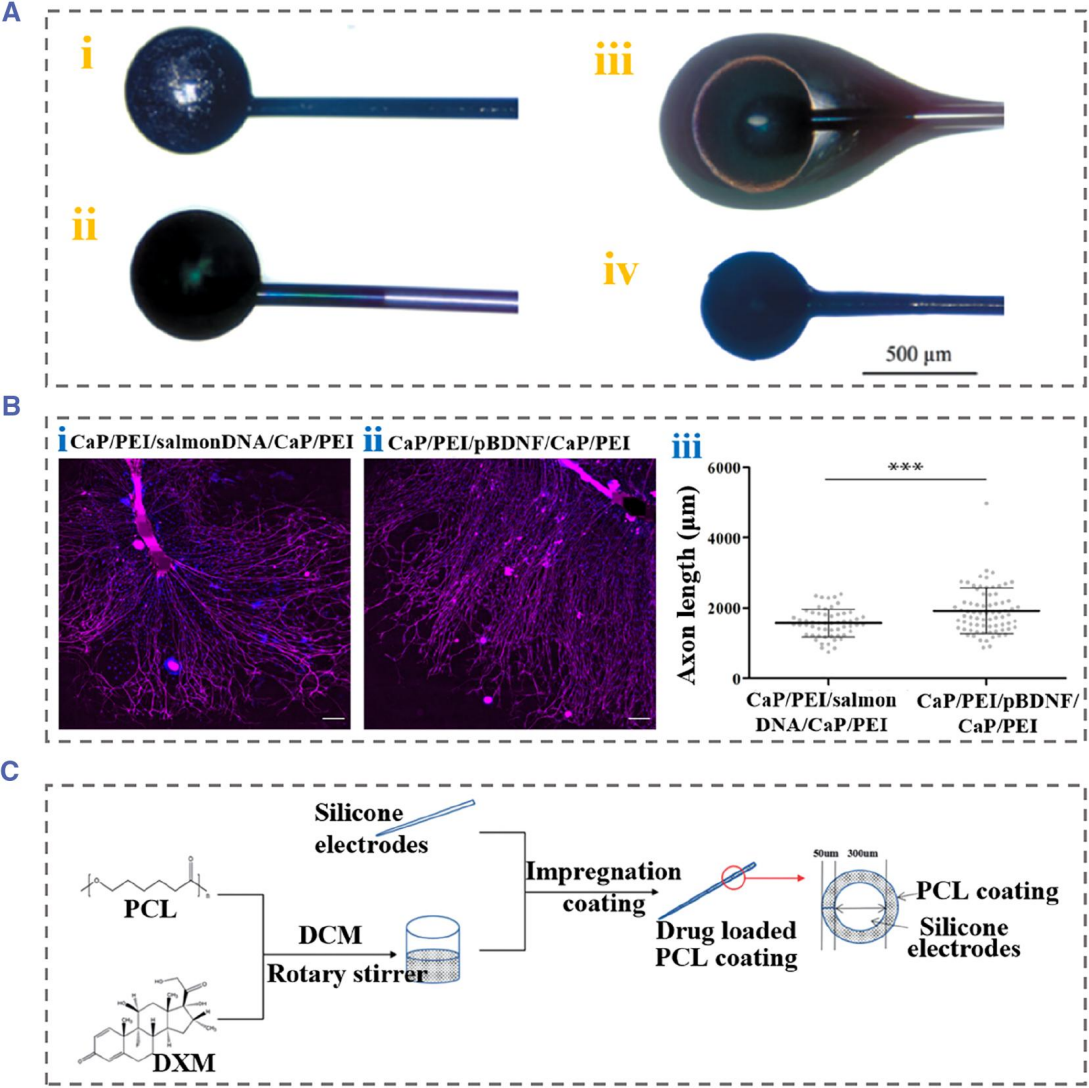
(A) Bare cochlear electrode (i) and cochlear electrode coated with conductive PEDOT (ii), RGD‐alginate hydrogel and PEDOT (iii), and dehydrated RGD‐alginate hydrogel and PEDOT (iv). Reproduced with permission.^76^ Copyright 2012, Elsevier. (B) (i–ii) Immunofluorescence images of SGN explants that were treated with control nanoparticles (i) or nanoparticles carring BDNF (ii), scale bars represent 200 μm in (i–ii). (iii) Axon length of SGN explants treated with different nanoparticles. Reproduced with permission.^77^ Copyright 2021, Elsevier. (C) Schematic diagram of the fabrication of DXM/PCL cochlear electrode coatings. Reproduced under terms of the CC‐BY license.^78^ Copyright 2021, The Authors, published by Informa UK Limited. BDNF, brain derived neurotrophic factor; DXM, dexamethasone; PCL, poly‐e‐caprolactone; PEDOT, poly (3, 4‐ethylenedioxythiophene); SGN, spiral ganglion neuron.

Recently, Hassarati et al. reported that coating a conductive hydrogel significantly enhanced the electrical properties of implantable electrodes without adverse effects on their mechanical properties.^73^ Meanwhile, the conductive hydrogel coating showed a low impedance standard in electrical testing. Several studies have also suggested that conductive hydrogel coating could lead neural cells to attach the electrode more commonly and stimulate cell growth compared to bare Pt electrode arrays.^74^, ^75^ However, there are no in vivo tests to prove their utility. Chikar et al. constructed a dual‐component CI coating that included alginate hydrogel functionalized with arginine‐glycine‐aspartic acid (RGD), neurotrophic factor, and conductive polymer (Figure 6A).^76^ Their results demonstrated that the modified CI resulted in the reduced impedance, promoted charge transfer, and released brain derived neurotrophic factor into the cochlea, indicating a great improvement in device performance after the above coating. In another study by Wey et al., a CI electrode coated with calcium phosphate (CaP) nanoparticles modified by DNA encoding neurotrophic factors was fabricated through the layer‐by‐layer technique.^77^ To prevent the degradation of alien DNA by nuclease in vivo, the nanoparticles were prepared by precipitation method, and polyethylene imine or carboxymethyl cellulose was used as the substrate to stabilize DNA.^79^ It was suggested that the electrode coating with nanoparticles carrying nucleic acids promoted the outgrowth of SGNs, thereby improving the transmission of electrical pulses (Figure 6B). Unfortunately, the evaluation of the feasibility of this electrode coating has not been carried out in vivo implantation studies. Luo et al. fabricated a new cochlear electrode coating using poly‐e‐caprolactone (PCL) carrying dexamethasone (DXM) to avoid cochlear fibrosis caused by CI implantation (Figure 6C).^78^ Specifically, 0%, 5%, 10%, and 20% DXM were added to different ratios of PCL mixtures with high and low molecular weights. Their results suggested that the prepared PCL coating had good biocompatibility, safe and slow degradability, great drug‐loading capability and adjustable thickness, contributing to controllable drug delivery.

### 4.3 Electrical stimulation

**FIGURE 7:**
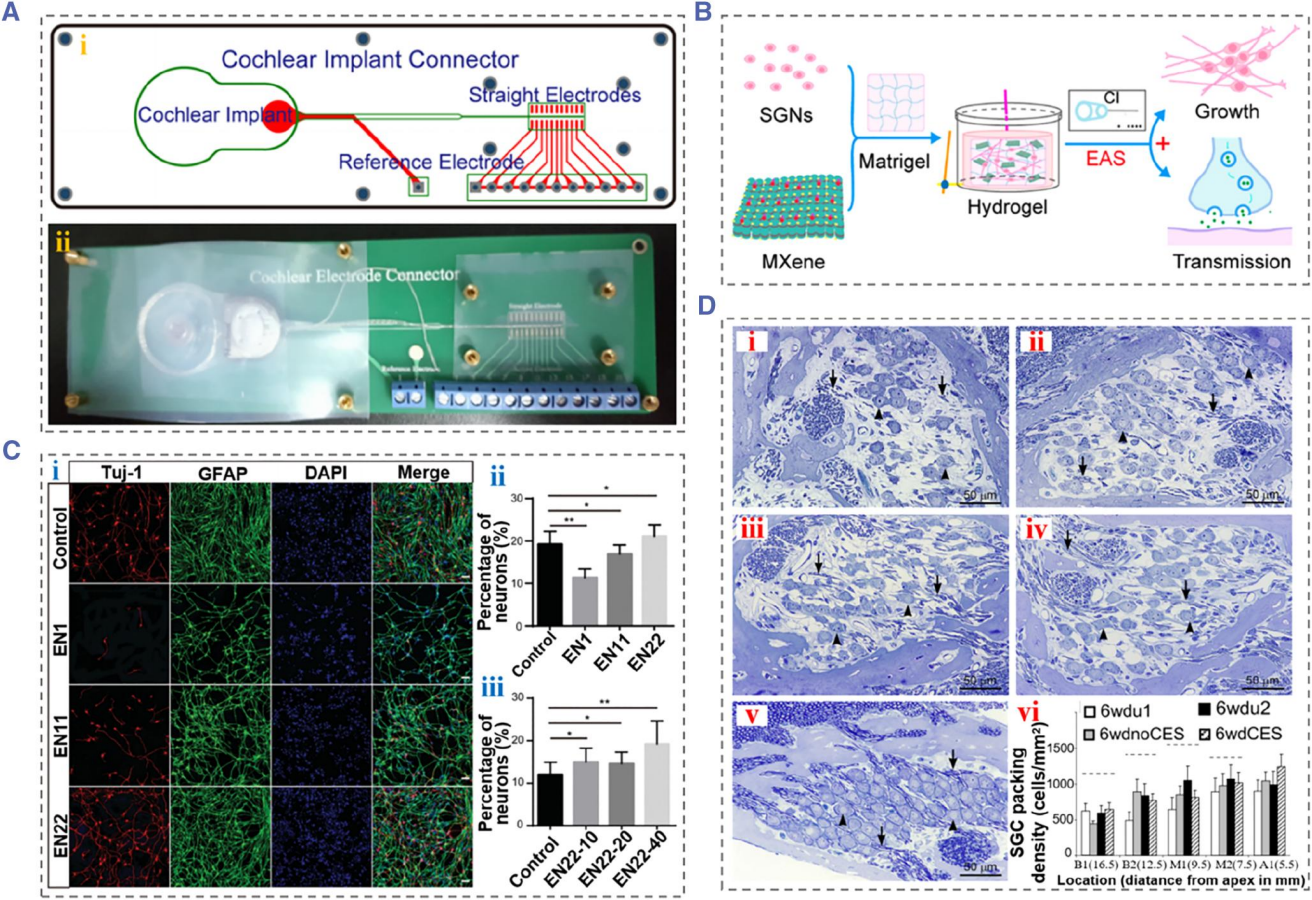
(A) Schematic diagram (i) and photograph (ii) of the printed circuit board connected with CI. Reproduced with permission.^88^ Copyright 2019, American Chemical Society. (B) Schematic diagram of the fabrication of 3D electrical stimulation culture system based on CI and T_i_3C_2_T_x_ MXene‐Matrigel hydrogel and its regulation on SGNs. Reproduced with permission.^89^ Copyright 2022, The Authors, published by American Chemical Society. (C) Immunofluorescence images of NSCs induced by the lower frequency electric‐acoustic stimulation produced by CI/graphene EAS system. EN 1, 11, 22 referred to high‐, middle‐, and low‐frequency EAS severally. EN22‐10, EN22‐20, and EN22‐40 represented EAS with EN22 for 10, 20, 40 min per day, respectively. Reproduced with permission.^90^ Copyright 2021, Royal Society of Chemistry. (D) (i–v) Optical images of Rosenthal's canal at the cochlear location. (vi) The average of SGN densities at different locations. Reproduced with permission.^91^ Copyright 2010, Elsevier. CI, cochlear implant; EAS, electric‐acoustic stimulation; EN, electrode number; NSCs, neural stem cells; SGN, spiral ganglion neuron.

CI bypasses most of the peripheral auditory system and HCs to directly stimulate residual SGNs to achieve hearing, which is of great significance for patients with sensorineural deafness hearing loss.^80^, ^81^, ^82^ Thus, the survival and normal function of SGNs are major factors influencing the effectiveness of cochlear implantation. Besides, it is essential to understand the regulatory effects and mechanisms of electrical stimulation on the survival, morphology and function of SGNs. Several studies have shown that electrical stimulation has a protective effect on SGNs.^83^, ^84^, ^85^, ^86^, ^87^ Researchers have explored the effects of different modes of electrical stimulation on cochlear cells and hearing function. Our team has designed a biocompatible electric‐acoustic stimulation (EAS) system based on CI to provide the electrical stimulation on cells, as shown in Figure 7A, and investigated the effects of the constructed CI/graphene EAS system on the behaviors of SGNs.^88^ Briefly, SGNs were seeded on graphene substrates and subjected to electrical stimulation transformed from CI. It was suggested that prolonged electrical stimulation promoted the neurite elongation of SGNs, which might be related to the promoted growth cone development. Furthermore, Liao et al. established a 3D electrical stimulation culture system by combining CI and Ti_3_C_2_T_x_ MXene‐Matrigel hydrogel. Their results demonstrated that low‐frequency electrical stimulation promoted the neurite extension of SGNs and signal transmission between cells (Figure 7B).^89^ In another study, Guo et al. further used the CI/graphene EAS system to culture neural stem cells (NSCs) and applied electrical stimulation transduced by sound waves detected by CI.^90^ Their results showed that EAS with higher frequency led to the death of NSCs, while lower frequency EAS promoted the proliferation and neuronal differentiation of NSCs (Figure 7C). This study highlights the potential of the CI‐based EAS system in stem cell therapy for hearing loss.

In addition, some studies have explored the influences of electrical stimulation of CI on SGN survival through in vivo experiments. Martijn et al. investigated whether chronic electrical stimulation patterns in CI users (amplitude modulated, high pulse rate) affected the survival, morphology and function of SGNs in guinea pigs with hearing loss. The results suggested that the chronic electrical stimulation had no effect on the densities, perikaryal area, and cell circularity of SGNs and did not affect the electrically evoked auditory brainstem responses thresholds and amplitudes of suprathreshold eABRs (Figure 7D).^91^

### 4.4 Bionic scaffolds

**FIGURE 8:**
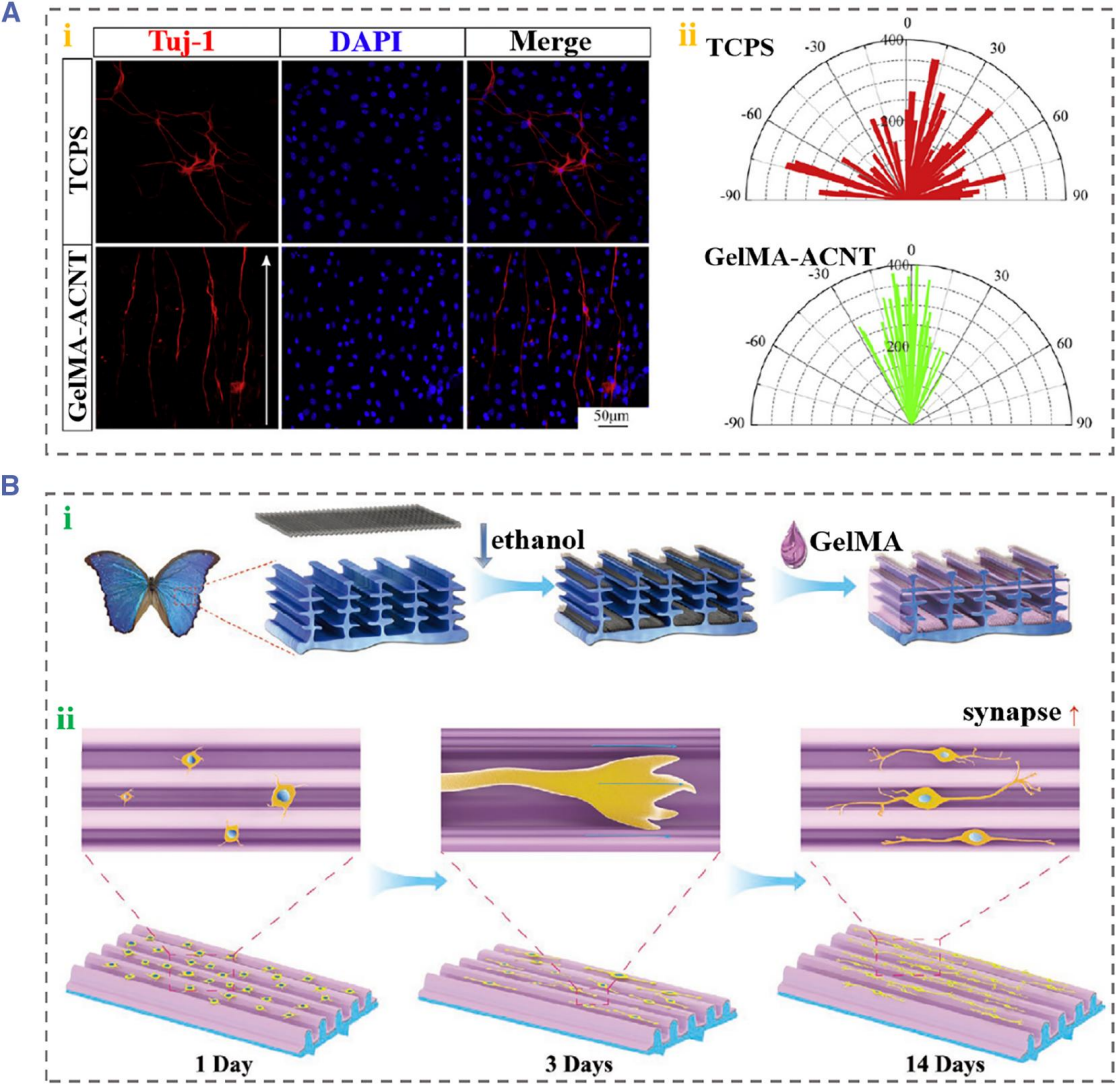
(A) (i) Immunofluorescence images of SGNs on different substrates. Tuj‐1 (red) is a marker of neurons and 9 DAPI (blue) represents the nuclei of cells. (ii) Polar histograms of neurite distribution on different substrates. Reproduced with permission.^97^ Copyright 2022, Elsevier. (B) (i) The preparation diagram of the SA‐CNT/GelMA‐modified wings. (ii) Growth and extension of SGNs on the modified wings. Reproduced with permission.^98^ Copyright 2021, John Wiley and Sons. DAPI, 2‐(4‐Amidinophenyl)‐6‐indolecarbamidine dihydrochloride; SA‐CNT, super‐aligned carbon‐nanotube sheets; SGN, spiral ganglion neuron.

The apoptosis and degeneration of SGNs can directly lead to the impairment of the auditory pathway, resulting in serious hearing disorders, and the directed regeneration of SGNs has exhibited promise in the treatment of hearing loss.^92^ Recently, scaffolds for guiding neural behaviors have been constructed using various biomaterials to promote nerve regeneration.^93^, ^94^, ^95^, ^96^ Hu et al. reported a conductive and anisotropic substrate by assembling super‐aligned carbon‐nanotube sheets (SA‐CNTs) onto biocompatible GelMA hydrogel (GelMA‐ACNT) (Figure 8A).^97^ The composite substrate inherited the conductivity and topological morphology of SA‐CNTs and the good cytocompatibility of GelMA hydrogel. The authors used the GelMA‐ACNT as substrates to culture SGNs, and the results suggested that the composite substrates boosted the growth cones, thereby conducive to the growth and maturation of SGNs. Furthermore, SGNs cultured on GelMA‐ACNT well displayed orientation and formed anisotropic neuronal networks with the guidance of the surface topology of the composite substrates. In addition, as shown in Figure 8B, Wei et al. presented a novel type of anisotropic substrate by combining SA‐CNTs and *Morpho menelaus* butterfly wing for the culture and orientation of SGNs.^98^ The topological substrates were fabricated by modifying SA‐CNTs on *M. menelaus* butterfly wing and covering them with GelMA hydrogel. The constructed SA‐CNTs‐modified wings promoted the neurite growth and cellular arrangement of SGNs. Additionally, the substrates facilitated the synaptic maturation, which was significant to the connections of the neural network. Their results demonstrated that the *M. menelaus* butterfly wing‐based conductive substrates contributed to the regeneration of auditory nerves. It was indicated that the construction of bionic scaffolds that combine various cues, including topographical cues and biochemical cues, is of great importance for auditory nerve regeneration.

## 5 CONCLUSION AND OUTLOOK

In this review, we give a summarization of the applications of biotechnologies and biomedical engineering technologies in the treatment of hearing loss. In detail, gene therapy, cochlear organoids, inner ear drug delivery, CI electrode coating, electrical stimulation and bionic scaffolds have been depicted. These methods exhibit great potential in the reconstruction of hearing, especially the hearing impairment due to the degeneration and loss of HCs and SGNs. However, there are some limitations to various approaches for hearing loss treatment.

For biological methods, gene therapy based on AAVs meets the therapeutic needs to a certain extent, but poor specificity and low infection efficiency hinder their development in treating hearing loss. The future and multidisciplinary research is necessary to screen out the practical AAV vector to improve specific and adult infection efficiency. Additionally, due to the complexity of the inner ear, the organoid construction is still tricky. We believe that the connection of the cochlea organoid and vestibular organoid using microfluidic systems is feasible. Based on microfluidic technology, the physiological and mechanical functions of the human body can be reproduced, and dynamic models can be built by precisely controlling the fluid flow, the mechanical signals and the interfaces of various tissues, thus realizing a more simulated model than the traditional static cell culture. If inner ear organoids or connected mini‐organs are successfully constructed in vitro, whether they are safely and effectively transplanted into the body and play biological functions can be further investigated, which will be a great breakthrough in the field of hearing disease research.

Despite the development of tissue engineering technology, the research and application of tissue engineering in the repair and regeneration of auditory injury is still nascent. As we have seen, numerous methods for inner ear drug delivery have been attempted, but improvements to the delivery system are still necessary. Biocompatibility of delivery systems is very important because they have the possibility to cause inflammation at the located site. Moreover, an in‐depth understanding of the final fate of delivery systems and the carried drugs is needed, especially for the system composed of non‐degradable materials. As for CI electrode coatings, delivering drugs via different systems during CI implantation to inhibit the inflammatory response and tissue fibrosis in the cochlea after implantation is currently a hot spot of research. However, most of the current studies showed rapid and unrepeatable drug delivery behaviors. Therefore, in the future, it is a promising direction to develop delivery systems with long‐term and repeatable delivery effects and perfectly integrate with CI electrodes. The inner ear of mammalian is an organ with a complex structure, and the cochlea is crucial for the production of hearing. The HCs and SGNs in the cochlea are closely connected functional cells with a well‐defined division of labor. Therefore, the key to repairing hearing loss is to induce the regeneration of HCs and SGNs and establish synaptic connections between them. However, the research on auditory nerve regeneration through engineering methods such as bionic scaffolds is currently mainly in vitro, and future in vivo experiments are necessary to validate these results.

## AUTHOR CONTRIBUTIONS

Renjie Chai, Tian Wang, Tao Xin, and Huan Wang provided the idea; Yangnan Hu, Le Fang, Hui Zhang, Shasha Zheng, Menghui Liao, Qingyue Cui, Hao Wei, Danqi Wu, Hong Cheng, Yanru Qi, and Renjie Chai wrote and revised the manuscript.

## CONFLICT OF INTEREST STATEMENT

The authors declare that there are no competing interests.
